# Risk Factors Associated with the Onset of Relapsing-Remitting and Primary Progressive Multiple Sclerosis: A Systematic Review

**DOI:** 10.1155/2015/817238

**Published:** 2015-01-31

**Authors:** Kyla A. McKay, Vivian Kwan, Thomas Duggan, Helen Tremlett

**Affiliations:** Faculty of Medicine (Neurology), Vancouver Coastal Health Research Institute, Brain Research Centre, University of British Columbia, Vancouver, BC, Canada V6T 2B5

## Abstract

Multiple sclerosis (MS) is a chronic central nervous system disease with a highly heterogeneous course. The aetiology of MS is not well understood but is likely a combination of both genetic and environmental factors. Approximately 85% of patients present with relapsing-remitting MS (RRMS), while 10–15% present with primary progressive MS (PPMS). PPMS is associated with an older onset age, a different sex ratio, and a considerably more rapid disease progression relative to RRMS. We systematically reviewed the literature to identify modifiable risk factors that may be associated with these different clinical courses. We performed a search of six databases and integrated twenty observational studies into a descriptive review. Exposure to Epstein-Barr virus (EBV) appeared to increase the risk of RRMS, but its association with PPMS was less clear. Other infections, such as human herpesvirus-6 and chlamydia pneumoniae, were not consistently associated with a specific disease course nor was cigarette smoking. Despite the vast literature examining risk factors for the development of MS, relatively few studies reported findings by disease course. This review exposes a gap in our understanding of the risk factors associated with the onset of PPMS, our current knowledge being predominated by relapsing-onset MS.

## 1. Introduction

Multiple sclerosis (MS) is a chronic central nervous system disease, characterized by demyelination and axonal loss. These physiological irregularities translate into significant clinical disability. Globally, an estimated 2.5 million people are affected [1], with the highest incidence and prevalence reported in Europe and North America [2, 3].

The clinical course of MS is highly heterogeneous. Approximately 85% of patients present with a relapsing-remitting course (RRMS), which is characterized by episodes of acute worsening of function followed by partial or complete recovery [4]. RRMS onset typically occurs in early adulthood, and, within around two decades, approximately half will go on to develop secondary progressive MS (SPMS) [5]. SPMS is defined as a steady clinical deterioration, independent of relapses [6]. Primary progressive MS (PPMS) affects only 10–15% of the MS population and is associated with a rapid disease progression [7]. The median age of onset is 40 years for progressive-onset (PPMS) and 30 years for relapsing-onset MS (RRMS/SPMS), a difference of ten years [7]. A higher female preponderance is consistently seen in relapsing-onset but not in progressive-onset MS [8].

The aetiology of MS is not well understood, but it is likely multifactorial, combining both genetic and environmental factors. The literature on the risk factors for MS has grown substantially in recent years, with best evidence to date indicating that a combination of a genetic predisposition, exposure to Epstein-Barr virus, cigarette smoking, and reduced sunlight exposure/vitamin D levels is involved [9]. Despite these advances, few studies have distinguished between the risk factors for relapsing-onset and progressive-onset disease. In view of the considerable differences in clinical presentation and prognosis, it is possible that these disease courses have distinctive risk factors. Through a systematic review of the literature, we aimed to identify and compare the known modifiable risk factors associated with the different clinical courses: relapsing-onset MS and progressive-onset MS.

## 2. Methods

This systematic review was based on a centralized protocol for Canada's National Population Health Study of Neurological Conditions, originally created by the University of Ottawa [10]. The protocol was adapted to study MS for the purposes of this systematic review.

### 2.1. Inclusion and Exclusion Criteria

All observational studies (case-control or cohort), systematic reviews, and meta-analyses examining at least one risk factor associated with the onset of multiple sclerosis were considered for inclusion. Studies must have reported results separately for one or both disease courses, namely, relapsing-onset (RR or SPMS) or progressive-onset (PP or progressive-relapsing MS). Progressive relapsing MS (PRMS) is a term used relatively infrequently, which describes a disease course in which patients experience a progressive-onset, later accompanied by occasional superimposed relapses [4], possibly affecting up to 28% of progressive-onset patients [11]. In this review, we will refer to the more common term PPMS to mean progressive-onset (PPMS or PR). Analyses that did not differentiate relapsing-onset from progressive-onset MS or who combined the progressive diseases into a single category (SPMS and PPMS/PRMS) were excluded. Additionally, studies must have reported a quantifiable measure of risk, involved human subjects, and be published in English to be included. No restriction was imposed in terms of age, sex, race, geographical residence, or source of population (e.g., community, hospital, outpatient, registry, or health administrative data).

Articles were excluded if they did not assess risk factors (e.g., studies looking at biomarkers for diagnosis) or did not present quantitative data (e.g., descriptive studies). As the focus of the review was on potentially modifiable risk factors, genetic and biological risk factors (e.g., sex and age) were not considered.

The primary outcome of interest was the onset of relapsing-remitting or primary progressive MS. Preferably, MS diagnosis and determination of the MS disease course would involve internationally recognized criteria, such as Schumacher, Poser, or McDonald criteria [12–15] for diagnosis and Lublin et al. [4, 16] for disease course, although studies using other methods (e.g., health administrative data or self-report for diagnosis) or local clinical judgment for disease course were considered. There is a large body of research describing the risk of reaching a diagnosis of MS in subgroups of patients with early clinical signs of MS, for example, those with optic neuritis or other clinically isolated syndromes. These studies typically focus on relapsing-onset MS only (by definition, PPMS does not present as CIS) and were considered beyond the scope of this review and were not included.

### 2.2. Search Methods to Identify Studies

The search for relevant publications was carried out in two stages; the first capitalized on a search strategy already in place which had focused on studies published up until 2012. These were included in a systematic review which examined the risk factors for MS onset and progression [9]. The results of this systematic review were searched and studies that reported findings by disease course were extracted. The second component of this systematic review was to update the original search to June 2014. The search strategy for both stages was developed using MeSH terms in MEDLINE (OvidSP) as well as relevant keywords. The term “multiple sclerosis” was exploded and searched in the title and/or abstract. The term “risk” was exploded and the terms “risk or etiology or genetic” were searched in the title and/or abstract. Relevant articles were initially identified from the following databases (the start year of each database is shown in brackets): MEDLINE (1996), EMBASE (1986), AGELINE (1982), CINAHL (1982), PSCYINFO (1990), and Cochrane Central Register of Controlled Trials (1991). The full search strategy can be viewed in the Appendix (see Tables 1–6 in Supplementary Material available online at http://dx.doi.org/10.1155/2015/817238).

### 2.3. Study Selection

Study selection was carried out in three stages (screening, quality assessment, and data extraction) by at least two independent reviewers. Any disagreement was resolved by discussion between the two reviewers, with a third reviewer consulted if needed.

Firstly, title and abstract screening were conducted, followed by evaluation of relevant full-text studies based on the inclusion criteria. Further, the citation lists of all included articles were scanned for any articles that may have been missed. Quality of the full-text articles was assessed using a prevalidated tool, the “Modified Downs and Black criteria” for observational studies [37]. Data were independently extracted by a single reviewer (VK) using a data collection form and were then checked by a second reviewer (KM) for accuracy.

## 3. Results

Of 122 studies published prior to 2012 which examined the risk of MS onset [9], only 15 specified the disease course when reporting findings. Five studies published since 2012 also met the inclusion criteria. A total of 20 original articles were included; 13 focused on infections, 2 on drug exposure (antibiotics or oral contraceptives), 3 on cigarette smoking, 1 on pregnancy, and 1 on month of birth. The median quality assessment score was 17 (range: 12–20), the maximum possible range being 0 to 20. No systematic reviews or meta-analyses met the inclusion criteria. Details of each study can be found in Table 1.

### 3.1. Viruses

Five studies were found which examined the association between Epstein-Barr virus (EBV) and risk of developing relapsing or progressive-onset MS [17–21].

As a component of a five-year longitudinal MRI study based in the UK, the sera of 25 RRMS and 25 PPMS patients were analyzed for EBV activity [17]. Significantly increased median titers of anti-Epstein-Barr virus nuclear antigen 1 (EBNA-1) IgG were found in RRMS compared to PPMS (670 versus 267 U/mL, *P* < 0.001). The opposite was true for median levels of EBV viral capsid antigen (VCA), which were lower in RRMS compared to PPMS (297 versus 530 U/mL, *P* < 0.05) [17].

Antibody levels against EBV were compared between 46 RRMS, 11 SPMS, and 21 PPMS patients in Iran [18]. All cases were prevalent, and disease duration at sample collection was not reported. Seroprevalence to anti-EBV IgG levels was significantly higher among RRMS (93.5%) and SPMS (100.0%) compared to PPMS (81.0%, *P* < 0.001 for both comparisons). Further, RRMS (15.2%) and SPMS (36.4%) showed more anti-EBV IgM reactivation than PPMS (0%, *P* < 0.001 for both comparisons) [18].

The US Army and Navy Physical Disability Agency records were searched for cases of MS and linked to the USA's Department of Defense Serum Repository (DoDSR) [19]. Serum samples collected prior to MS symptom onset were compared to age, sex, and ethnicity-matched controls from the DoDSR for activity to EBV. An increased risk of RRMS was associated with a 4-fold increase in anti-EBNA1 IgG serum antibody titers (RR: 2.3; 1.7–3.2) based on 122 cases and 234 controls. An increased risk of RRMS was also associated with a 4-fold increase in anti-EBNA complex serum antibody titers (RR: 3.3; 2.3–4.7) based on 164 cases and 315 controls [19].

A case-control study based in Spain examined the levels of EBV and human herpesvirus-6 (HHV-6) in the sera of RRMS (*n* = 49), SPMS (*n* = 49), and age and sex-matched healthy controls (*n* = 50) [20]. All MS patients had been enrolled in a clinical trial for MS but either were in the placebo arm or had not yet received the intervention at the time of study. Controls were volunteers or healthy donors (exact source was not stated). RRMS patients had higher levels of anti-HHV-6 IgM antibody titers than healthy controls (15.02 versus 10.19, *P* = 0.002). The authors reported “no significant differences” between RRMS, SPMS, and healthy controls for anti-EBV EBNA IgG or anti-CP antibodies (no *P* values given) [20].

A higher proportion of pediatric RRMS patients were seropositive for remote EBV infection from mouth swabs (19/22) compared to healthy age and sex-matched controls (35/77, *P* = 0.008) in a Canadian clinic-based study [21]. The mean disease duration of the MS patients was 2.5 years at baseline in this longitudinal study. Seropositivity rates to human herpesvirus-6 (HHV-6), human herpesvirus-7 (HHV-7), cytomegalovirus (CMV), and herpes simplex virus (HSV) did not differ between groups (*P* > 0.05 for all) [21].

Three studies were found which examined the association between human Herpesvirus (HHV-6) and risk of developing relapsing or progressive-onset MS [22–24].

A Jordanian case-control study reported no association between HHV-6 DNA and the risk of relapsing-onset MS [22]. Of 24 *β*-globin positive relapsing-remitting patients, 6 (reported as 24% by the authors) had a prior exposure to HHV-6, measured by the presence of the viral DNA in the serum compared to 8/33 (24.2%) who tested positive in the control group (30 healthy controls and 3 other neurological disease controls). The one individual with PPMS was HHV-6 DNA negative [22].

The presence of HHV-6 in peripheral blood mononuclear cells of RRMS, PPMS, and healthy and other neurological disease controls was examined in a Canadian clinic-based study [23]. HHV-6 DNA was detected in 20.8% (5/24) of controls and 23.0% (6/26) of RRMS patients (*P* > 0.05). Of the two PPMS patients included, both were seronegative for HHV-6 [23].

Sera from 22 RRMS patients and 66 controls from the USA's National Institute of Health (NIH) were analyzed for activity to HHV-6 [24]. HHV-6 early antigen IgM levels were higher in RRMS patients compared to controls (*P* < 0.0011), but there was no significant difference in IgG levels between groups [24].

Varicella zoster virus (VZV) is a member of the herpes-simplex family, which manifests clinically as chicken pox. History of VZV infection was measured by self-report in 65 RRMS patients, 23 PPMS patients, and 157 healthy or neurological disease controls in Mexico City [25]. The risk of RRMS was increased in people with a history of VZV infection (OR: 3.89; 2.05–7.36) when compared to healthy and neurological disease controls. The odds ratio was also elevated for PPMS, but it did not reach statistical significance in this smaller cohort (OR: 1.26; 0.52–3.03) [25].

Torque teno virus is a common virus that generally effects young children but is not currently known to be related to any specific disease symptomology [38]. It has been shown to increase production of proinflammatory cytokines and thus was investigated for its role in MS [39]. Serum and cerebrospinal fluid (CSF) samples were obtained from 104 RRMS, 31 PPMS, and 93 healthy controls from Italy [26]. Levels of TTV viremia were significantly lower in RRMS patients compared to healthy controls (4.6 versus 5.4 log^10^ copies/mL, *P* < 0.0001) [26]. PPMS patients had significantly higher levels than the RRMS patients (5.8 versus 4.6 log^10^ copies/mL, *P* = 0.0008) [26].

### 3.2. Chlamydia Pneumoniae (CP)

Using data from the prospective Nurse's Health Studies (NHS and NHSII), sera from 90 incident cases of RRMS and 180 healthy controls were analyzed for previous CP infection [27]. Of the MS cases included, eleven had their serum collected prior to the onset of MS. After adjustment for age, smoking history, latitude of residence at birth, and ancestry, prior infection with CP was not associated with a significantly increased risk of RRMS (1.7; 0.9–3.2). However, when progressive MS patients (SPMS or PPMS, *n* = 32) were included, MS risk was associated with CP seropositivity (OR: 1.7, 1.1–2.7).

The same research group later studied US Army personnel and exclusively used samples collected prior to the onset of disease for 47 RRMS, 15 PPMS, and 166 age, sex, race, and date of blood collection-matched controls. They found no association between CP and the risk of RRMS (OR: 0.8; 0.4–1.7) or PPMS (OR: 1.0; 0.3–3.7) when compared to healthy controls adjusting for latitude of residence at time of entry into active duty and education level [28].

Ninety-four prevalent relapsing-onset MS patients and 63 controls with other inflammatory neurological disease (OIND) and other noninflammatory neurological disease (ONIND) were assessed for seropositivity to CP in an Austrian case-control study. There was no statistically significant difference in CP seropositivity between any of the groups (RRMS 59.1%, SPMS 46.4%, OIND 64.1%, and ONIND 75.0%) [29].

### 3.3. Antibiotic Exposure

Antibiotic exposure in the three years prior to the onset of MS (or equivalent index date for controls) was examined among 163 MS patients and 1523 controls from the UK's General Practice Research Database [30]. A lower risk of RRMS was reported in people exposed to penicillin for more than 2 weeks relative to nonexposure (OR: 0.4; 0.2–0.8). There was not a significantly altered risk of PPMS associated with penicillin exposure (OR: 1.2; 0.3–4.9); however, the authors note that they could not make definitive conclusions due to the small number of PPMS patients included in the analysis (*n* = 18) [30].

### 3.4. Oral Contraceptive Use

A nested case-control study within the UK's General Practice Research Database examined the effect of recent oral contraceptive (OC) use on MS risk [31]. A modest protective effect against RRMS was reported for any OC use compared to non-use (OR: 0.6; 0.4–1.0) based on 97 RRMS cases and 1001 controls. There was no altered risk of PPMS (OR: 1.2; 0.2–6.7), though the number of cases was small (*n* = 9) [31].

### 3.5. Cigarette Smoking

The association between cigarette smoking and MS was assessed in a nested case-control study which used the UK's General Practice Research Database [32]. Smoking status was determined from medical record review and 159 RRMS and 20 PPMS patients were included in the study (*N* = 20 PPMS patients reported as included in the study (Table 1), but *n* = 22 PPMS patients reported along with the study findings in Table 2 of the original paper [32]). There was no altered risk of RRMS (OR: 1.3; 0.9–1.8) or PPMS (OR: 1.3; 0.5–3.1) in ever smokers versus never smokers [32].

A cohort study which included 102 clinic- and population-based PPMS and 443 RRMS patients in the UK assessed the risk of disease onset in ever smokers versus never smokers, based on self-report [33]. Smoking status did not alter the risk of developing PPMS compared to RRMS (OR: 0.82; 0.52–1.29) [33].

A Swedish study included a questionnaire asking 122 people with MS about their history of smoking [34]. Although a number of findings were reported, we were able to find one which separated the phenotypes, involving a subgroup of primary progressive (*n* = 17) and relapsing-onset MS patients (*n* = 58). Of 29 ever smokers who began smoking before the age of 15, 11 (38%) had a progressive onset compared to 6 of 46 never smokers (13%) (*P* = 0.012). By inference, the corresponding results for the relapsing onset patients were 18/29 (62%) early smokers versus 40/46 (87%) never smokers [34].

### 3.6. Pregnancy

The risk for RRMS was lower in parous compared to nulliparous women (74 actual nulliparous women versus 50.9 expected, *P* = 0.000009) in a study which included 153 Swedish women [35]. The risk of PPMS was not reported [35].

### 3.7. Month of Birth

In a Canadian study which included 14,799 MS patients (3,334 PPMS and 11,465 RRMS/SPMS), there was a significant difference in the birth ratios (May/November births) for relapsing-onset patients (Birth ratio: 1.43) compared to population controls (BR: 1.18) (*P* = 0.000032) [36]. Driving this difference is the nadir of births during November in the RRMS group compared to the population control group (6.7 versus 7.7%, *P* = 0.000076). No month of birth effect was found for the PPMS group. The study, however, reported an unusually high proportion of PPMS patients (22.5%) [36].

## 4. Discussion

We systematically reviewed the literature on risk factors for the development of relapsing-onset and primary progressive MS, with a focus on modifiable factors. The vast majority (18/20) of articles reported on risk factors for relapsing-onset MS, and although just over half (12/20) examined risk factors associated with progressive-onset MS, a high proportion (5/12) was only able to compare the risk relative to relapsing-onset MS. Further, the progressive-onset groups were invariably much smaller in number than the relapsing-onset (in keeping with the epidemiology of PPMS). While these observations are not unexpected, our review highlights how little is known about risk factors associated with the onset of PPMS; our current knowledge being predominated by relapsing-onset MS which may or may not have the same aetiology as PPMS.

The involvement of viruses in the aetiology of MS has long been a subject of controversy. To date, no single virus has been established as the causal factor for MS; however, certain viral infections likely increase susceptibility to MS [40]. There is considerable evidence linking the Epstein-Barr virus (EBV) to MS in general [41]. Best evidence suggested that Epstein-Barr virus (EBV) was associated with an increased risk of RRMS [19, 21], but its association with PPMS was less clear. RRMS patients tended to have higher antibodies to EBV than PPMS patients [17, 18]. HHV-6 exposure did not appear to be related to the risk of RRMS (versus healthy controls) in 3 of 5 studies [22–24], although IgM antibody levels to the virus were higher in prevalent RR cases (versus control) in two studies [20, 24]. In the one study able to explore the association between HHV-6 and PPMS, no differences relative to either RRMS or “healthy” controls could be found (at the *P* = 0.05 level), but this was based on a small cohort (*n* = 14 PPMS) [24]. Chlamydia pneumoniae (CP) is a ubiquitous bacteria and a common cause of pneumonia. From the three studies identified from our review, chlamydia pneumoniae infection did not appear to be related to the onset of either RRMS [27–29] or PPMS [29]; two of these studies were able to obtain serum samples pre-MS onset [27, 28].

Despite extensive research supporting a role for cigarette smoking and the risk of MS, with 14 studies included in a recent systematic review [42], we found just one study where the actual risk of developing MS was reported separately for the MS disease phenotypes [32] and two studies where the relative risk of developing one disease phenotype over another was reported [33, 34]. The former study found no altered risk of developing either RRMS or PPMS in “ever versus never” smokers in a UK general population (although the PPMS cohort was small, comprising of 20 cases only (*N* = 20 PPMS patients reported as included in the study (Table 1), but *n* = 22 PPMS patients reported along with the study findings in Table 2 of the original paper)) [32]. The two latter studies examined the smoking exposure differently, making them challenging to compare, one found smoking (ever versus never) not to be associated with disease phenotype and was able to include 102 individuals with PPMS [33] and the other found a preponderance of PPMS (versus RRMS) associated with early adolescent smoking, although it was based on just 17 PPMS patients [34]. Oral contraceptive use and penicillin exposure were both associated with a reduced risk of RRMS [30, 31]. While neither exposure was significantly associated with PPMS risk (based on 9 [31] and 18 [30] PPMS patients, resp.), it was of interest to note that the estimated odds ratios for PPMS risk were in an opposite direction compared to relapsing-onset MS in both studies [30, 31].

One study was found which considered pregnancy and onset of RRMS; the risk of RRMS was reported as reduced in parous (versus nulliparous) women [35]. We were unable to find a pregnancy-related study for PPMS. Another study reported that the month of birth affected later risk of developing RRMS but not PPMS [36]. However, this study also included an increased proportion of individuals with PPMS (22.5% of the entire cohort) which appears to be higher than the expected 10–15% reported in most cohorts [43]. Further, although a number of studies have reported a month of birth effect in MS (without differentiating disease course) [44], a recent reevaluation of the methodology employed indicated that all of these findings might be spurious [45].

A major strength of this review is its systematic methodology, which employed a comprehensive search strategy and accessed multiple databases. All articles retrieved from this search were screened at three levels (title, abstract, and full paper) by multiple trained researchers to ensure that relevant articles were not missed. Generally, the studies included were of high quality with the median quality assessment score of 17 out of a maximum possible score of 20.

The scientific literature on MS is large and ever increasing; therefore, it is possible that some articles were still missed. Publication bias was a possibility we could not avoid; researchers may be less likely to complete and submit a “negative” study finding and Editors might be less likely to accept these papers [46]. Many of the studies relied on prevalent cases of MS (13/20) and thus could not collect information on the exposure prior to disease onset. As a result, it was possible that some of the exposures under study may have actually occurred after disease onset or have been a consequence of the disease itself or have been susceptible to recall bias.

A large number of articles were excluded from this review because the study authors did not specify the disease course or did not report findings by disease course separately. For those studies where the disease course was known, this could represent a missed opportunity. We recognize there could be many reasons that authors chose not to evaluate their results by disease course—including reliability of disease course information. Further, adequate statistical power to assess the risk of PPMS is difficult to achieve due to its low prevalence. Only 4 of the 12 studies assessing PPMS risk included more than 25 such individuals each. Consequently, authors often noted that they were wary of drawing definitive conclusions based on such small sample sizes. Given the relatively low prevalence of PPMS, a consortium type effort may be needed in order to access a large enough cohort of PPMS patients.

In summary, this systematic review exposes a gap in our understanding of the risk factors for the different clinical disease courses of MS. To date, most studies have combined disease courses (or not differentiated them); hence the current understanding of MS risk largely relates to the predominant disease course (RRMS), as these individuals far outnumber those with PPMS. Although we cannot conclude from this review that there are differences in the risk factors driving the two main MS disease phenotypes, it remains a possibility. When possible, future studies should consider disease course when examining risk factors for MS to allow for a more comprehensive understanding of this complex disease.

## Supplementary Material

The supplementary material contains the detailed search strategy that was employed in six online databases (MEDLINE, EMBASE, AgeLine, CINAHL, PsycINFO, and Cochrane Central Register of Controlled Trials). This review capitalized on a previous systematic review which captured articles published prior to 2012; therefore this search exclusively encompassed articles published after 2012.

## Figures and Tables

**Table 1:** Main characteristics of studies examining risk factors for relapsing-onset or primary progressive multiple sclerosis.

Systematic Review of Risk Factors for relapsing and progressive onset MS												
Author, Year, Location	Study design	Cases/controls	Disease course (number of subjects)		Source of cases	Source of controls	Sex of cases (M/F)	Main risk factors	Incident or prevalent cases (disease duration at time of main exposure	Main findings/statistics		Quality assessment^†^
			Relapsing onset	Progressive onset					(i.e., risk factor) for prevalent cases)	Relapsing-onset (RRMS or SPMS^**^)	Progressive-onset (PPMS or PRMS)	
Farrell et al. [17], 2009, United Kingdom	Mixed-original cohort study with healthy controls included later	100/20	CIS (50)^*^ RRMS (25)	PPMS (25)	University hospital	Source not stated; healthy controls^*^	42/58	Epstein-Barr virus (EBV) Varicella Zoster virus (VZV)	Prevalent (longitudinal study with repeated measures; disease duration at point of exposure measurement not clear)	For RR versus PPMS: higher median EBNA-1 IgG (670 versus 267 U/mL, *P* < 0.001) and lower EBV VCA titer levels (297 versus 530 U/mL, *P* < 0.05)		17

Ramroodi et al. [18], 2013, Iran	Case-control	78/123	RRMS (46) SPMS (11)	PPMS (21)	University hospital	Medical laboratory; healthy blood donors	22/56	Epstein-Barr virus (EBV)	Prevalent (disease duration not reported)	PPMS had lower seroprevalence of EBV IgG (81.0%) than RRMS (93.5%) and SPMS (100%) (*P* < 0.001 for both). SPMS (36.4%) and RRMS (15.2%) showed more anti-EBV-IgM reactivation than PPMS (0%)		15

Munger et al. [19], 2011, USA	Nested case-control	**EBNA-1** 122 RRMS/ 234 controls **EBNAc** 164 RRMS/ 315 controls	RRMS (167)	Progressive MS (3)^*^ Other (23)^*^	US Army and Navy Physical Disability Agency and the Department of Defense Serum Repository	Department of Defense Serum Repository	148/74	Epstein-Barr virus (EBV)	Incident	Risk of developing RRMS increased with a 4-fold increase in average anti-EBNA-1 IgG titers (RR: 2.3, 1.7–3.2) and anti-EBNA complex titers (RR: 3.3, 2.3–4.7)	NR	18

Villoslada et al. [20], 2003, Spain	Case-control	151/50	CIS (53)^*^ RRMS (49) SPMS (49)	None	Clinical trials	Source not stated; volunteers and healthy donors	33/65	Epstein-Barr virus (EBV) Human herpesvirus-6 (HHV-6) Chlamydia pneumoniae (CP)	Prevalent (mean disease duration = 1.8 years [RRMS]; 15.9 years [SPMS])	RRMS patients had higher levels of anti-HHV-6 IgM antibody titers than healthy controls (15.02 versus 10.19, *P* = 0.002). No statistically significant difference between RRMS, SPMS, and healthy controls for anti-EBV EBNA IgG or anti-CP antibodies	NA	18

Yea et al. [21], 2013, Canada	Case-control	22/77	Pediatric RRMS (22)	None	Clinic-based	Community advertisement; healthy controls	6/16	Epstein-barr virus (EBV)	Prevalent (mean disease duration = 2.5 years)	Higher proportion of RRMS patients were seropositive for remote EBV infection (19/22) compared to healthy age and sex-matched controls (35/77, *P* = 0.008)	NA	17

Ahram et al. [22], 2009, Jordan	Case- control	36/37	RRMS (30) (only 24 included in analysis) SPMS (5)	PPMS (1)	Clinic-based	Clinic-based; healthy controls (34) and other neurological disease (3)	11/25	Human herpesvirus-6 (HHV-6)	Prevalent (disease duration not reported)	24% (6/24) of RRMS patients, 40% (2/5) of SPMS patients, and 24.2% (8/33) of controls were HHV-6 positive. No statistically significant difference in the presence of HHV-6 viral DNA between any of the groups (*P* > 0.05)	Insufficient sample size (*n* = 1)	14

Mayne et al. [23], 1998, Canada	Case-control	40/24	RRMS (32) (only 26 included in analysis) SPMS (12)	PPMS (2)	Multicentre clinic-based	Source not stated; healthy controls and patients with other neurological illness	16/30	Human herpesvirus-6 (HHV-6)	Prevalent (disease duration not reported)	No association between HHV-6 infection and RRMS; HHV-6 DNA detected in (5/24) 20.8% of controls and (6/26) 23% of RRMS patients *P* > 0.05)	Insufficient sample size (*n* = 2)	12

Soldan et al. [24], 1997, USA	Case-control	36/66 (controls: 14 healthy controls, 31 OND, and 21 OID)	RRMS (22)	PPMS (14)	Clinic-based	Clinic-based; healthy controls and OND or OID	NR	Human herpesvirus-6 (HHV-6)	Prevalent (disease duration not reported)	Increased IgM response to early antigen of HHV-6 in RRMS compared to healthy controls (*P* < 0.001). No differences in IgG response among any groups tested	No differences in IgG or IgM response in PPMS versus controls (*P* = 0.06) or RRMS (*P* > 0.05)	13

Rodríguez-Violante et al. [25], 2009, Mexico	Case-control	126/157	RRMS (65) SPMS (38)	PPMS (23)	Clinic-based	Source not stated; healthy controls and patients with OND	40/86	Varicella virus (VZV)	Prevalent (disease duration not reported)	Previous VZV infection significantly associated with RRMS (OR: 3.89; 2.05–7.36) and SPMS (OR: 2.98; 1.4–6.3)	Association not found in PPMS (OR: 1.26; 0.52–3.03)	13

Mancuso et al. [26], 2013, Italy	Case-control	207/93	RRMS (104) SPMS (48)^*^ Benign (24)^*^	PPMS (31)	Unclear	Source not stated; healthy controls	66/141	Torque teno virus	Prevalent (disease duration not reported)	Lower TTV viremia in RRMS compared to healthy controls (4.6 versus 5.4 log⁡(10) copies/mL, *P* < 0.0001)	Higher TTV viremia in PPMS compared to RRMS (5.8 versus 4.6 log⁡(10) copies/mL, *P* = 0.0008)	14

Munger et al., [27] 2003, USA	Nested case-control	141/282	RRMS (90) SPMS or PPMS (32)^*^ Unknown (19)^*^	SPMS or PPMS (32)^*^ Unknown (19)^*^	USA's Nurse's Health Study	USA's Nurse's Health Study; healthy controls	0/141	Chlamydia pneumoniae (CP)	Incident	Chlamydia pneumoniae antibody seropositivity not associated with risk of RRMS (OR: 1.7; 0.9–3.2)	NR (SPMS and PPMS were analyzed together)	20

Munger et al. [28], 2004, USA	Nested case-control	129/258	**Army** RRMS (47) SPMS (3)^*^ Unknown (18)^*^ **KPMCP** RRMS (7) SPMS OR PPMS (26)^*^ Unknown (13)^*^	**Army** PPMS (15) Unknown (18)^*^ **KPMCP** SPMS OR PPMS (26)^*^ Unknown (13)^*^	US Army Physical Disability Agency Database and the Kaiser Permanente Northern California Health Plan (KPMCP)	US Army Physical Disability Agency Database and the Kaiser Permanente Northern California Health Plan (KPMCP); healthy controls	60/69	Chlamydia pneumoniae (CP)	Incident	CP seropositivity did not predict risk of RRMS (OR: 0.8; 0.4–1.7). Fourfold difference in titer levels of anti-CP IgG antibodies did not increase risk for RR course at onset (OR: 0.9; 0.6–1.3)	CP seropositivity did not predict risk of PPMS (OR: 1.0; 0.3–3.7). Fourfold difference in titer levels of anti-CP IgG antibodies did not increase risk for PPMS course at onset (OR: 1.1; 0.6–2.0)	20

Krametter et al. [29], 2001, Austria	Case-control	94/63	RRMS (66) SPMS (28)	None	Source not stated	Source not stated: patients with OND	37/57	Chlamydia pneumoniae (CP)	Prevalent (mean disease duration = 4.7 years [RRMS]; 8.7 years [SPMS])	Seropositivity to CP was not significantly different between the RRMS/SPMS patients and controls. Seroprevalence of: **IgG** (RRMS 59.1%, SPMS 46.4%, OIND 64.1%, ONIND 75.0%), **IgM** (RRMS 10.6%, SPMS 3.6%, OIND 5.1%, ONIND 4.2%), **IgA** (RRMS 54.5%, SPMS 57.1%, OIND 61.5%, ONIND 70.8%)	NA	15

Alonso et al. [30], 2006,United Kingdom	Nested case-control	163/1523	RR onset (145)	PPMS (18)	General Practice Research Database	General Practice Research Database	NR	Antibiotic use	Incident	Risk of RRMS was reduced with more than 2 weeks of penicillin use compared to no use in 3 years prior to onset (OR: 0.4, 0.2–0.8)	No altered risk of developing PPMS with more than 2 weeks of penicillin use compared to no use in 3 years prior to onset (OR: 1.2, 0.3–4.9)	17

Alonso et al. [31], 2005, United Kingdom	Nested case-control	106/1001	RRMS (87) SPMS (10)	PPMS (9)	General Practice Research Database	General Practice Research Database	0/106	Oral contraception (OC) use	Incident	RRMS risk was modestly reduced in OC users compared to nonusers (OR: 0.6; 03–0.9)	PPMS risk was not influenced by OC use when comparing any OC users to nonusers (OR: 1.2; 0.2–6.7)	18

Hernán et al. [32], 2005, USA	Nested case-control	201/1913	RRMS (159) SPMS (22)	PPMS (20)	General Practice Research Database	General Practice Research Database; healthy controls	60/141	Cigarette smoking	Incident	No altered risk of RRMS in ever smokers [*n* = 81] versus never smokers [*n* = 98] (OR: 1.3; 0.9–1.8) [179/181 relapsing-onset MS included in this analysis]	No altered risk of PPMS in ever smokers versus never smokers (OR: 1.3; 0.5–3.1).^□^	18

Manouchehrinia et al. [33], 2013, United Kingdom	Cohort	895	RRMS (443) SPMS (350)	PPMS (102)	Clinic and population-based; estimated to capture >98% local geographical region	NA	270/625	Cigarette smoking	Prevalent (mean disease duration = 17 years)	Smoking status (ever versus never) was not associated with risk for onset of PPMS compared to RRMS (OR: 0.82; 0.52–1.29)		18

Sundström and Nyström [34], 2008, Sweden	Cohort	122	RRMS 96	PPMS (26)	Epidemiological survey	NA	44/78	Cigarette smoking	Prevalent (median disease duration = 6 years)	Of the ever smokers starting before the age of 15 (*n* = 29); 11 (38%) had a progressive onset versus 6 of the 46 never smokers (13%) (*P* = 0.012). [note: only a subgroup were included in the analysis: 17 progressive onsets and 58 relapsing onsets].		14

Runmarker and Andersen [35], 1995, Sweden	Cohort	153 (cases)	108 RRMS/SPMS	45 PPMS	Hospital outpatients	NA	0/153	Pregnancy	Incident	The risk for RRMS was lower in parous compared to nulliparous women (74 actual nulliparous women versus 50.9 expected, *P* = 0.000009)	NR	19

Sadovnick et al. [36], 2007, Canada	Case-control	14,799 MS cases 13,783 unaffected siblings 13675451 population controls	RRMS/SPMS (11,465)	PPMS (3334)	Canadian Collaborative Project on Genetic Susceptibility to MS	Unaffected siblings and Canadian population controls	Not reported	Month of birth	Prevalent (disease duration not reported)	Birth ratio (May/November) was higher for RRMS compared to controls (1.43 versus 1.18, *P* = 0.000032)	Birth ratio (May/November) was not significantly different for PPMS compared to controls (1.15 versus 1.18, *P* > 0.05)	18

CIS = clinically isolated syndrome.

NA = not applicable.

NR = not reported.

OID = other inflammatory disease.

OND = other neurological disease.

PPMS = primary progressive multiple sclerosis.

RRMS = relapsing-remitting multiple sclerosis.

SPMS = secondary progressive multiple sclerosis.

^*^These individuals did not contribute to the main findings/statistics reported in the final columns.

^**^CIS patients not included, unless otherwise stated.

^□^22 PPMS patients were included in the analysis (Table  2 from original paper) despite *n* =20 PPMS patients reportedly included in the overall study.

^†^Quality assessment was based on the Modified Downs and Black criteria for observational studies [37]. The assigned score could range from 0 to 20; a higher score implies better quality.
