# Supplementary material for: Risk Factors Associated with the Onset of Relapsing-Remitting and Primary Progressive Multiple Sclerosis: A Systematic Review
Source: Biomed Res Int. 2015 Jan 31;2015:817238. doi: 10.1155/2015/817238 (PMC4329850; doi:10.1155/2015/817238)
Supplement: Supplementary file 1 — The supplementary material contains the detailed search strategy that was employed in six online databases (MEDLINE, EMBASE, AgeLine, CINAHL, PsycINFO, and Cochrane Central Register of Controlled Trials). This review capitalized on a previous systematic review which captured articles published prior to 2012; therefore this search exclusively encompassed articles published after 2012. [file 817238.f1.pdf]

Appendix

Search strategies for online databases

This supplementary material contains the detailed search strategy that was employed in six online databases (MEDLINE, EMBASE, AgeLine, CINAHL, PsycINFO, and Cochrane Central Register of Controlled Trials). This review capitalized on a previous systematic review which captured articles published prior to 2012; therefore this search exclusively encompassed articles published after 2012.

Table 1. Search strategy for MEDLINE (OvidSP)

1. exp multiple sclerosis/ or exp multiple sclerosis, chronic progressive/ or exp multiple sclerosis, relapsing-remitting/
2. multiple sclerosis.tw.
3. exp risk/
4. prevention & control.fs.
5. (risk or etiology or genetic*).ti,ab.
6. etiology.fs.
7. Genetics.fs.
8. 3 or 4 or 5 or 6 or 7
9. Epidemiologic Studies/
10. Exp Case-control studies/
11. case control.tw.
12. exp Cohort Studies/
13. Cohort analy$.tw.
14. (cohort adj (study or studies)).tw.
15. (Follow up adj (study or studies)).tw.
16. (observational adj (study or studies)).tw.
17. 9 or 10 or 11 or 12 or 13 or 14 or 15 or 16
18. 1 or 2
19. 8 and 17 and 18
20. limit 19 to yr="2012-Current"

Table 2. Search strategy for EMBASE (OvidSP)

1. exp multiple sclerosis/
2. multiple sclerosis.tw.
3. exp risk factor/
4. pc.fs.
5. (risk or etiology or genetic*).ti,ab.
6. Et.fs.
7. Ep.fs.

8. exp epidemiology/
9. exp case control study/
10. case control.tw.
11. exp cohort analysis/
12. Cohort analy$.tw.
13. (cohort adj (study or studies)).tw.
14. (follow up adj (study or studies)).tw.
15. (observational adj (study or studies)).tw.
16. 1 or 2
17. 3 or 4 or 5 or 6 or 7
18. 8 or 9 or 10 or 11 or 12 or 13 or 14 or 15
19. 16 and 17 and 18
20. limit 19 to yr="2012-Current"

Table 3. Search strategy for AgeLine (EBSCO)

1. "Multiple Sclerosis"
2. Limit to 2012-2014

Table 4. Search strategy for CINAHL (EBSCO)

1. "Multiple Sclerosis"
2. "risk" or "etiolog*" or "genetic*"
3. "epidemiol*" or "cohort" or "observational"
4. 1 and 2 and 3
5. Limit to 2012-2014

Table 5. Search strategy for PsycINFO (EBSCO)

1. "Multiple Sclerosis"
2. "risk" or "etiolog*" or "genetic*"
3. "epidemiol*" or "cohort" or "observational"
4. 1 and 2 and 3
5. Limit to 2012-2014

Table 6. Search strategy for Cochrane Central Register of Controlled Trials (EBSCO)

1. Multiple sclerosis.mp. [mp=title, short title, abstract, full text, keywords, caption text]
2. Limit 1 to yr= "2012-Current"
